# Insulin resistance and muscle insulin receptor substrate‐1 serine hyperphosphorylation

**DOI:** 10.14814/phy2.12236

**Published:** 2014-12-03

**Authors:** Charles A. Stuart, Mary E. A. Howell, Brian M. Cartwright, Melanie P. McCurry, Michelle L. Lee, Michael W. Ramsey, Michael H. Stone

**Affiliations:** 1 Department of Internal Medicine, Quillen College of Medicine, East Tennessee State University, Johnson City, Tennessee; 2 Department of Allied Health, College of Clinical and Rehabilitative Health, East Tennessee State University, Johnson City, Tennessee; 3 Department of Exercise and Sports Science, Clemmer College of Education, East Tennessee State University, Johnson City, Tennessee

**Keywords:** Insulin receptor substrate‐1, insulin resistance, metabolic syndrome, muscle

## Abstract

Insulin resistance in metabolic syndrome subjects is profound in spite of muscle insulin receptor and insulin‐responsive glucose transporter (GLUT4) expression being nearly normal. Insulin receptor tyrosine kinase phosphorylation of insulin receptor substrate‐1 (IRS‐1) at Tyr896 is a necessary step in insulin stimulation of translocation of GLUT4 to the cell surface. Serine phosphorylation of IRS‐1 by some kinases diminishes insulin action in mice. We evaluated the phosphorylation status of muscle IRS‐1 in 33 subjects with the metabolic syndrome and seventeen lean controls. Each underwent euglycemic insulin clamps and a thigh muscle biopsy before and after 8 weeks of either strength or endurance training. Muscle IRS‐1 phosphorylation at six sites was quantified by immunoblots. Metabolic syndrome muscle IRS‐1 had excess phosphorylation at Ser337 and Ser636 but not at Ser307, Ser789, or Ser1101. Ser337 is a target for phosphorylation by glycogen synthase kinase 3 (GSK3) and Ser636 is phosphorylated by c‐Jun N‐terminal kinase 1 (JNK1). Exercise training without weight loss did not change the IRS‐1 serine phosphorylation. These data suggest that baseline hyperphosphorylation of at least two key serines within muscle IRS‐1 diminishes the transmission of the insulin signal and thereby decreases the insulin‐stimulated translocation of GLUT4. Excess fasting phosphorylation of muscle IRS‐1 at Ser636 may be a major cause of the insulin resistance seen in obesity and might prevent improvement in insulin responsiveness when exercise training is not accompanied by weight loss.

## Abstract (precis)

Insulin resistance can be induced in mice by several different kinases that phosphorylate insulin receptor substrate‐1 on any one of a few specific serines. Immunoblots of muscle from 33 subjects with the metabolic syndrome showed excess basal phosphorylation at serines 337 and 636 compared with muscle from 17 lean controls, suggesting involvement of glycogen synthase kinase‐3 (GSK3) and c‐Jun N‐terminal kinase 1 (JNK1). Normal phosphorylation at other tested serines indicated that inhibitory kappa B kinase beta (IKKβ), AMP‐dependent kinase (AMPK), and protein kinase C theta (PKCθ) were not directly implicated in these subjects.

## Background

After decades with a stable prevalence of obesity in the United States, there has been a steady increase in adult obesity rates beginning the 1980s (Flegal et al. 2010). The percentage of Americans who are obese more than doubled between 1980 and 2000. The prevalence of diagnosed diabetes increased by more than 150% over the same period (Polonsky 2012). More than half of all adults are now overweight (BMI > 25 kg/m^2^), with some regions of the United States having in excess of two‐thirds of the population being overweight or obese (BMI > 30 kg/m^2^). Because of obesity‐related illness, the average life expectancy in the United States may soon decline for the first time (Bray and Bellanger 2006). The preventable excess obesity‐related mortality is projected to soon exceed that associated with cigarette smoking. Insulin resistance and hyperinsulinemia are key elements of the Metabolic Syndrome that is marked by visceral obesity, hypertension, hyperlipidemia, hyperglycemia, and coronary heart disease (Grundy et al. 2004; Kahn et al. 2005). The metabolic syndrome is considered to be prediabetes and effective preventive measures are urgently being sought (Kahn et al. 2005). The mechanism of the severe insulin resistance in the metabolic syndrome remains unclear.

The next intracellular step in insulin action after activation of the cell surface insulin receptor tyrosine kinase is phosphorylation of insulin receptor substrate‐1 (IRS‐1) on a specific tyrosine residue (Saltiel and Kahn 2001). However, there are many additional sites in IRS‐1 that are phosphorylated by several other intracellular serine/threonine protein kinases.

Studies of insulin receptor substrate‐1 (IRS‐1) phosphorylation in mice have demonstrated a status of chronic low‐grade inflammation associated with adipose tissue cytokine production in obesity (McCurdy and Klemm 2013). The IKK*β*/NF*κ*B inflammatory cascade has been shown to cause phosphorylation of IRS‐1 at Ser307 and diminish insulin signaling (Gao et al. 2002). Cytokine activation of the JAK STAT pathway is associated with IRS‐1 phosphorylation at Ser636 and Ser307 (Johnston et al. 2003). Activation of mTOR and S6K1 is also associated with phosphorylation of Ser636 and Ser307. Strength training exerts its effect on muscle fiber hypertrophy through activation of mTOR and resultant phosphorylation of downstream targets (Frost and Lang 2011), but mTOR‐mediated phosphorylation of IRS‐1 Ser636 inhibits insulin action in mice (Ozes et al. 2001).

These studies of IRS‐1 phosphorylation in mice suggest that the obesity of the metabolic syndrome might result in insulin resistance via obesity‐related chronic inflammation and activation of the JNK1 pathway and the IKK‐NFkB cascade causing serine phosphorylation of IRS‐1 at Ser307 and Ser636. Exercise training could modify the basal IRS‐1 phosphorylation status at Ser636 and/or Ser789 via activation of mTOR and AMPK. The studies reported here evaluated the phosphorylation status by immunoblots of muscle IRS‐1 at key serines in untrained metabolic syndrome subjects and quantified those again post training.

## Research design and Methods

### Subject selection

Fifty subjects participated in this study. Subjects with diabetes were excluded. Thirty‐three subjects deemed to be at high risk for diabetes were recruited meeting the entry criteria of obesity (BMI > 30 kg/m^2^) and a close family member having type 2 diabetes. The other 17 subjects were sedentary controls who were nonobese without a family history of diabetes. None of the subjects engaged in regular exercise for at least 1 year. All of the obese subjects met the criteria for metabolic syndrome (Kahn et al. 2005) because they also had waist circumference greater than 40 inches, insulin resistance documented by euglycemic clamp, and dyslipidemia (low HDL cholesterol, high triglycerides, or high LDL cholesterol). Each of the subjects provided written valid consent approved by the East Tennessee State University Institutional Review Board.

### The exercise intervention

Thirty‐two volunteers (including 22 metabolic syndrome subjects) participated in increasing intensity strength training without weight loss for 8 weeks as previously described (Layne et al. 2011). Eighteen additional participants (11 metabolic syndrome subjects) were subjected to increasing intensity and duration endurance training on stationary bicycles for 8 weeks with no weight loss permitted. The amount of energy expended each week by the subjects in each of the two exercise training protocols was closely matched throughout the 8 weeks based on time and intensity. Estimated exercise‐related energy expenditure began at 250 kcal per day and peaked at 450 kcal per day in week 7.

### Anthropometric assessments

Each subject underwent measurement of body composition and insulin responsiveness. A percutaneous muscle biopsy was performed just prior to the euglycemic clamp study.

#### Muscle biopsies

A small sample (100–150 mg) of thigh muscle was obtained. Percutaneous needle biopsies of vastus lateralis were performed after an overnight fast and 2 hours of quiet recumbency as previously described Stuart et al. (2006). The sample was immediately divided into two pieces, one of which was mounted on cork and frozen in an isopentane slurry and the other was frozen in liquid nitrogen for later processing.

#### Euglycemic hyperinsulinemic clamp

Immediately following the muscle biopsy, an insulin clamp was performed. After a 2‐hour baseline period, an insulin infusion at 40 mU/m^2^/min was performed for 2 hours to achieve a physiological insulin concentration of about 50 *μ*U/mL and a stable glucose infusion rate (SSGIR) to quantify insulin sensitivity (Reeds et al. 2006). Fasting serum glucose and insulin concentration were determined as part of the euglycemic clamp procedure.

#### Body composition

Body fat and lean body mass were measured by air displacement plethysmography (BodPod, Concord, CA).

### Quantification of muscle content of total and phosphorylated insulin receptor substrate‐1 (IRS‐1) by immunoblots

Muscle was homogenized and subjected to polyacrylamide gel electrophoresis through Tris‐acetate 3–8% gradient gels (Invitrogen, Carlsbad, CA) as previously described (Layne et al. 2011). After transfer to membranes and blocking with buffer containing bovine serum albumin (Cell Signaling, Danvers, MA), incubations overnight with primary antibodies directed against the epitope of interest were performed. Antibodies against IRS‐1 pSer337 (#2580) and pSer789 (#2389) were obtained from Cell Signaling. Antibodies were obtained from Abcam (Cambridge, MA) that were specific for total IRS‐1 (ab52167), pTyr896 (ab4873), pSer307 (ab1194), pSer636 (ab53038), and pSer1101 (ab55343). Second antibody with enhanced chemiluminescence using SuperSignal West Pico Chemiluminescence substrate (Pierce, Rockford, IL) was subsequently added and images were generated using a G‐BOX from Syngene (Frederick, MD). The relative intensity of the digital signal intensity was quantified using QuantityOne software from Bio‐Rad (Hercules, CA).

### Statistical calculations

All data are displayed as mean ± standard error, except as explicitly indicated. Comparing data between the two groups was performed using the independent t test except as noted. Pretraining and post‐training comparisons were made using the paired t test. Relationships between select variables were assessed using Pearson Product Moment Correlation. Statistical procedures were performed using SigmaPlot version 12.2 from Systat Software (San Jose, CA).

## Results

### Insulin resistance in metabolic syndrome subjects

All of the metabolic syndrome subjects were insulin resistant. The steady‐state glucose infusion rate (SSGIR) determined by the euglycemic insulin clamp studies averaged 38% of that of the sedentary control subjects (Table 1). Fasting insulin concentration was double that of the controls. Fasting serum glucose was higher in the metabolic syndrome group. Table 1 displays several other pertinent characteristics of the subjects with BMI, waist circumference, lean body mass, and fat mass all being greater in the metabolic subject group. VO_2_max was less than controls. Fasting triglycerides and LDL cholesterol were increased and HDL cholesterol was decreased in the metabolic syndrome subjects. Serum alkaline phosphatase (AP) was quantified as an indirect indicator of hepatic steatosis. Metabolic syndrome AP was increased, although serum AST and ALT hepatocellular enzymes were not increased (data not shown).

**Table 1.** Subject characteristics*

	Units	Sedentary controls	Metabolic syndrome	*P* value	Sedentary controls	Metabolic syndrome	*P* value	Sedentary controls	Metabolic syndrome	*P* value
Number of subjects		17	33		10	11		7	22	
Gender		10F/7M	11F/22M		Female	Female		Male	Male	
Age	Years	38 ± 3	43 ± 2	0.108	39 ± 4	44 ± 3	0.294	38 ± 4	43 ± 2	0.208
BMI	kg/m^2^	24.1 ± 0.8	34.8 ± 0.6	<0.001	23.7 ± 1.2	34.2 ± 0.8	<0.001	24.7 ± 1.0	35.1 ± 0.8	<0.001
Waist	cm	92 ± 3	114 ± 2	<0.001	90 ± 4	114 ± 2	<0.001	95 ± 4	115 ± 3	<0.001
Lean body mass	kg	47.3 ± 2.2	62.7 ± 1.9	<0.001	41.9 ± 1.3	50.5 ± 1.5	<0.001	56.3 ± 2.7	68.3 ± 1.5	<0.001
Fat mass	kg	20.5 ± 2.1	42.3 ± 1.6	<0.001	20.1 ± 2.4	41.9 ± 2.9	<0.001	21.2 ± 4.3	42.4 ± 2.0	<0.001
VO_2max_	mL/kg.min	31.0 ± 1.5	25.2 ± 0.8	<0.001	28.9 ± 2.2	21.2 ± 1.1	<0.001	33.6 ± 1.2	26.5 ± 0.7	<0.001
Fasting glucose	mmol/L	5.1 ± 0.2	5.7 ± 0.1	0.004	4.9 ± 0.2	5.5 ± 0.2	0.075	5.3 ± 0.3	5.8 ± 0.1	0.053
Fasting insulin	pmol/L	46 ± 7	94 ± 10	<0.001	42 ± 7	83 ± 11	<0.001	51 ± 14	100 ± 14	<0.001
Clamp final glucose	mmol/L	4.9 ± 0.1	5.0 ± 0.1	0.689	4.8 ± 0.1	4.9 ± 0.1	0.829	5.1 ± 0.2	5.1 ± 0.1	0.880
Clamp final insulin	pmol/L	362 ± 27	404 ± 31	0.274	355 ± 42	373 ± 38	0.520	370 ± 27	439 ± 49	0.278
Serum triglycerides	mg/dL	127 ± 22	171 ± 19	0.019	108 ± 20	196 ± 49	0.022	154 ± 44	158 ± 16	0.524
Serum HDL	mg/dL	50 ± 3	39 ± 1	0.002	57 ± 2	44 ± 2	<0.001	39 ± 4	37 ± 2	0.569
Serum LDL	mg/dL	94 ± 6	120 ± 6	0.008	91 ± 7	114 ± 12	0.192	98 ± 10	122 ± 7	0.073
Serum AP	U/L	58 ± 4	69 ± 3	0.025	58 ± 6	74 ± 5	0.060	57 ± 4	66 ± 3	0.161
SSGIR	mg/kg.min	6.04 ± 0.44	2.32 ± 0.19	<0.001	6.29 ± 0.67	2.20 ± 0.27	<0.001	5.68 ± 0.53	2.39 ± 0.25	<0.001

* data are displayed as mean ± standard error, SSGIR steady‐state glucose infusion rate.

### Muscle IRS‐1 pretraining baseline phosphorylation

**Figure 1.**
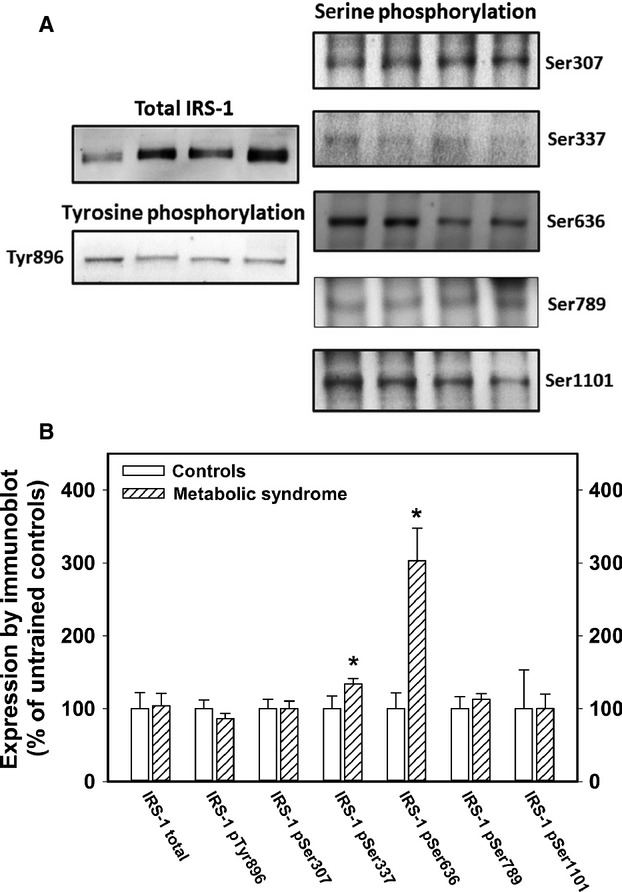
IRS‐1 content of muscle from metabolic syndrome subjects. (Panel A) shows sample immunoblots for each of the antibodies used for quantifying total IRS‐1 and phosphorylation at six specific sites. Each blot shown here had lanes containing samples from metabolic syndrome subjects alternating with samples from controls. The band on the left of each image shown is a metabolic syndrome subject. Panel B summarizes the data for each antibody. All of the data are expressed in relation to the mean of the untrained sedentary control subjects for each assay. The data displayed are the mean and standard errors from at least two separate assays for each subject. The number of subjects that had complete data in each assay was variable due to sample limitations. The data shown in Panel B represent 10–12 controls and 14–18 metabolic syndrome subjects. The asterisk indicates significant difference (*P* < 0.05, *t* test) from the control subject data for that assay.

The total IRS‐1 content and the amount of phosphorylated IRS‐1 at several specific sites on the molecule were determined using specific antibodies in immunoblots. Total IRS‐1 content of muscle was not different in the metabolic syndrome subjects as displayed in Fig. 1B. Phosphorylation of tyrosine at position 896 (Tyr896) was not different and the ratio of phosphorylation at Tyr896 to total IRS‐1 averaged 17% less in the metabolic syndrome, but this decrease in the ratio was not statistically significant. Phosphorylation at Ser307, Ser789, and Ser1101 did not differ between the groups, but the phosphorylation at Ser337 and Ser636 were significantly more in the metabolic syndrome group.

### The effect of exercise training of the excess phosphorylation of IRS‐1 at Ser337 and Ser636

The protocols of exercise training that were used in these studies did not alter the insulin responsiveness of metabolic syndrome subjects or controls as quantified by euglycemic clamp studies (metabolic syndrome post training 2.16 ± 0.28 mg/kg.min, *P* = 0.685; controls post training 6.85 ± 0.81 mg/kg.min, *P* = 0.354). Fasting insulin concentrations did not change either (metabolic syndrome post training 128 ± 28 pmol/L; controls post training 31 ± 4 pmol/L).

**Figure 2.**
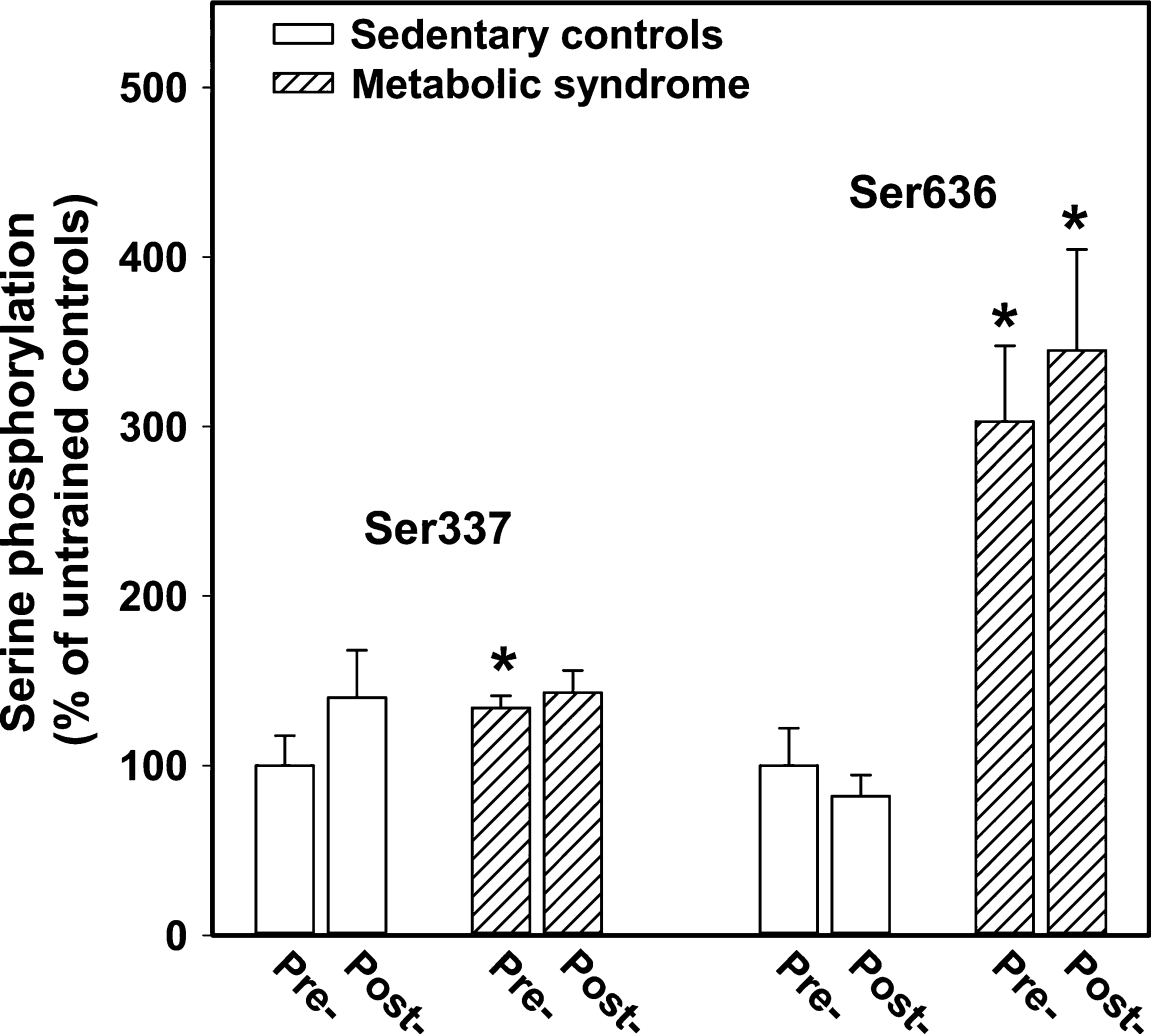
The effect of exercise training on muscle IRS‐1 phosphorylation at Ser337 and Ser636. Pre‐ and postexercise training data are displayed in this figure. Eight weeks of training, either progressive resistance or a stationary bike program, did not significantly alter the level of baseline phosphorylation of Ser337 or Ser636 as quantified by western blots. Shown here are the mean and standard errors of results of at least two separate experiments including pre‐ and posttraining muscle specimens from 12 controls and 18 metabolic syndrome subjects. The asterisk denotes significant difference (*P* < 0.05) from the corresponding control data. There was no significant increase or decrease in phosphorylation of Ser337 or Ser636 after exercise training in controls or metabolic syndrome subjects.

Exercise training for 8 weeks without weight loss did not change the fasting phosphorylation status of muscle IRS‐1 at Ser337 and Ser636 (Fig. 2). In both groups, there was a trend toward 10–20% increases in the phosphorylation status at Ser789, and Ser1101, but no change in phospho‐Ser307.

**Figure 3.**
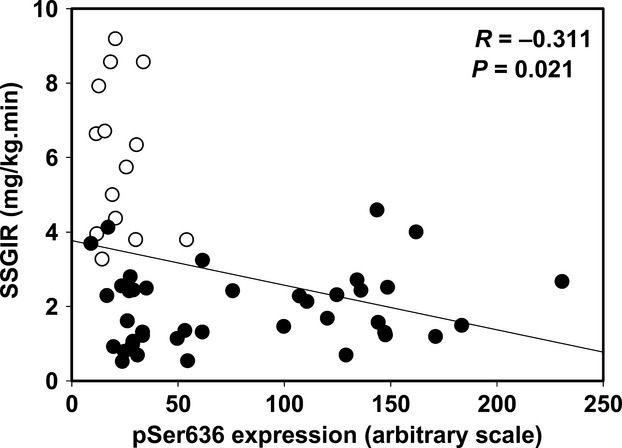
Correlation between insulin responsiveness and baseline phosphorylation at IRS‐1 serine 636. The data shown are the euglycemic clamp steady‐state glucose infusion rate (SSGIR) plotted against the relative amount of phosphorylation at IRS‐1 serine 636. The data shown are before and/or after training for 11 controls (open symbols) and 20 metabolic syndrome subjects (filled circles). The missing subjects had inadequate muscle left for this assay to be done. The correlation coefficient (R) and p value, calculated using Pearson Product Moment, are shown on the graph.

Phosphorylation at muscle IRS‐1 Ser636 inversely correlated with SSGIR as shown in Fig. 3. The data displayed in the figure include both pretraining and posttraining muscle biopsies. Analyzing the correlation in the controls alone or the metabolic syndrome subjects alone did not show a statistically significant correlation. There was considerable overlap between the controls and the metabolic syndrome subjects, but about half of the metabolic syndrome subjects had much higher Ser636 phosphorylation. The statistical correlation is dependent on including the control data. Indeed, this suggests that excess Ser636 phosphorylation may be important in some, but does not account for all of the insulin resistance among the metabolic syndrome subjects. The correlation of SSGIR with phosphorylation at Ser337 was significant only in the pretraining data (*R* = −0.398, *P* = 0.049, *n* = 25). Total IRS‐1, pTyr896, pSer307, pSer789, and pSer1101 did not correlate with SSGIR or fasting insulin concentration.

## Discussion

The insulin resistance of the metabolic syndrome subjects in this study was dramatic. The metabolic syndrome insulin responsiveness quantified by euglycemic insulin clamp studies was less than half that seen in age‐ and gender‐matched sedentary control subjects. Previous studies found that in spite of severe insulin resistance, muscle insulin receptor and GLUT4 expression were normal (Stuart et al. 2013). In fact, exercise interventions increased insulin receptor and GLUT4 expression but did not improve whole‐body insulin responsiveness, suggesting that an intermediate step in insulin stimulation of glucose uptake was impaired (Layne et al. 2011; Stuart et al. 2013). This study found that total IRS‐1 expression was not decreased in metabolic syndrome subjects, nor was baseline tyrosine phosphorylation at Tyr896. Immunoblots using antibodies directed at specific phosphorylated sites showed that the baseline phosphorylation status of serines at sites 337 and 636 were increased compared with control subjects.

JNK1 and mTOR‐S6K1 phosphorylate both Ser307 and Ser636 and a few others. GSK3 specifically phosphorylates IRS‐1 at Ser337. The IRS‐1 serine at position 789, a target for AMPK, was not different in metabolic syndrome subjects. Phosphorylations of IRS‐1 at Ser307 (a target of JNK and IKK) and Ser1101 (a target of PKC*θ*) were not increased in metabolic syndrome muscle. Exercise training for 8 weeks with no weight loss did not improve insulin responsiveness in euglycemic clamp studies and the baseline phosphorylation status of Ser337 and Ser636 in metabolic syndrome muscle was unchanged.

In skeletal muscle, IRS‐1 is phosphorylated at tyrosine 896 by the insulin‐activated tyrosine kinase within the beta subunit of the insulin receptor (Saltiel and Kahn 2001). Insulin and insulin‐like growth factor‐1 (IGF‐1) act as growth factors through their respective cell surface receptors via tyrosine phosphorylation of IRS‐1, which in turn associates the catalytic and regulatory subunits of phosphatidylinositol‐3 kinase (PI‐3K) resulting in the phosphorylation of phosphatidylinositol bis‐phosphate (PIP_2_), thereby generating PIP_3_, the principal intracellular mediator of insulin action (LeBrasseur et al. 2011). PIP_3_ causes assembly of Src and Akt on *β*‐arrestin‐2 scaffolding, activating Akt (Luan et al. 2009), at least one isoform of which is critical in GLUT4 translocation (Wilson and Rotwein 2007).

Phosphorylation of IRS‐1 at one or more of several key serines acts to terminate the insulin signal by ending the association of the PI‐3K subunits on IRS‐1. Insulin itself may eventually terminate its action on glucose uptake by phosphorylation of IRS‐1 at one or more serines by several kinases that are activated directly or indirectly by PIP_3_ (De and Roth 1997; Ozes et al. 2001; Liberman and Eldar‐Finkelman 2005). Additional kinases that mediate inflammation and stimulation of protein synthesis by insulin and other growth factors may play a role in terminating or limiting insulin‐stimulated PI‐3K activation. Activation of the protein synthetic muscle hypertrophy pathway by resistance training or growth factors involves phosphorylation and activation of the mammalian target of rapamycin (mTOR). IRS‐1 is phosphorylated at Ser636 by S6K1 which is activated by mTOR. This serine phosphorylation inactivates PI‐3K and limits insulin stimulation of protein synthesis by this negative feedback loop (De and Roth 1997; Ozes et al. 2001). Insulin stimulates glycogen synthase at least in part by decreasing its serine phosphorylation through inhibition of glycogen synthase kinase‐3 (GSK3). The apparent phosphorylation of IRS‐1 at Ser337 by GSK3 may also act as a negative feedback loop to turn off insulin‐stimulated activation of PI‐3K‐related stimulation of glucose uptake and glycogen synthesis (Liberman and Eldar‐Finkelman 2005). Nikoulina and coworkers have shown that the GSK3 activity was nearly doubled in skeletal muscle from type 2 diabetes subjects coincident with lower activity of glycogen synthase compared with lean and obese nondiabetic subjects (Nikoulina et al. 2000). They also found that higher GSK3 activity was associated with lower insulin‐stimulated glucose disposal rates during euglycemic clamps in their subjects.

JNK has also been demonstrated to act by phosphorylation of IRS‐1 at Ser636, a site that was robustly hyperphosphorylated in our metabolic syndrome subjects' muscle. In spite of 8 weeks of exercise training, the hyperphosphorylation at Ser636 was unchanged, which is consistent with the fact that the amount of adipose tissue in each subject was also unchanged after training.

Another potential site of IRS‐1 serine phosphorylation that could mediate insulin resistance was at Ser1101. Li and coworkers demonstrated that PKC*θ*‐mediated phosphorylation at Ser1101 inhibited insulin stimulation of glucose uptake (Li et al. 2004). Phosphorylation at Ser1101 inhibited tyrosine phosphorylation of IRS‐1 in response to insulin and thus effects downstream of IRS‐1 were diminished (Li et al. 2004). Our studies did not find excess phosphorylation at this site.

Direct evidence of IRS‐1 being the critical site of impaired insulin action in human obesity and type 2 diabetes has been elusive. Searches for abnormalities in IRS‐1 primary structure have identified three polymorphisms that affect 10–15% of patients with type 2 diabetes, but essentially the same prevalence of these substitutions was found in control subjects (Laakso et al. 1994; Sigal et al. 1996). The most frequent polymorphism was a G to A substitution in the gene sequence that caused a glycine to arginine amino acid change at codon 792 (Gly792Arg polymorphism). Hribal and coworkers overexpressed Arg^792^‐IRS‐1 in L6 skeletal muscle cells and demonstrated diminished insulin action including less PI‐3K activation, less phosphorylation of AKT, less translocation of GLUT1 and GLUT4, and less glucose uptake into the cells (Hribal et al. 2000). These data suggest a possible role of altered primary structure of IRS‐1 in developing the insulin resistance of the metabolic syndrome and type 2 diabetes is rare and does not contribute to the mechanisms of the vast majority of subjects with insulin resistance.

Studies in mice and in cell culture have shown multiple activated protein kinases phosphorylate IRS‐1 on serines that dampen the insulin signal (Boura‐Halfon and Zick 2009). Yi and coworkers have shown that insulin causes increased phosphorylation of at least six IRS‐1 serines and a decrease in phosphorylation of two serines and two threonines in muscle from normal subjects (Yi et al. 2007). The insulin resistance of obesity is at least partly due to adipocyte‐produced cytokines which circulate and interact with muscle cell surface JAK receptors (Johnston et al. 2003). Subsequent involvement of intracellular STAT and JNK systems have been shown directly or indirectly through mTOR/S6K1 to cause increased phosphorylation of multiple IRS‐1 serines (307, 337, 636, 1101). Our immunoblot data using antibodies against phospho‐serines found increased basal phosphorylation of Ser337 and Ser636, but no increase in phosphorylation at Ser307 or Ser1101. Ser636 hyperphosphorylation may be due to inflammation, activation of mTOR, or both. Excess phosphorylation of Ser337 suggests that GSK3 activation may also be involved in the inhibition of transmittal of the insulin signal downstream of IRS‐1.

The baseline hyperphosphorylation of IRS‐1 at Ser337 and Ser636 coincides with the diminished glucose uptake into the muscle in response to increments in blood insulin concentrations in many of the metabolic syndrome subjects. The higher significant negative correlation between insulin responsiveness and phosphorylation at Ser636 suggests that this site may be the more important of the two sites that had excess phosphorylation. Previous reports have shown that excess phosphorylation at Ser337 by GSK3 or at Ser636 by JNK1 and phospho‐mTOR can cause insulin resistance by preventing insulin receptor‐mediated tyrosine phosphorylation of IRS‐1 (Ozes et al. 2001; Liberman and Eldar‐Finkelman 2005). Our data showed only a small decrease in muscle IRS‐1 baseline Tyr896 phosphorylation in metabolic syndrome subjects suggesting, at least in the fasted situation, tyrosine phosphorylation was adequate (albeit with a more than two‐fold higher insulin concentration). We did not measure IRS‐1 tyrosine phosphorylation after the euglycemic clamp insulin infusion, but based on studies by other groups in mice (Aguirre et al. 2002; Boura‐Halfon and Zick 2009), we infer that insulin‐stimulated phosphorylation of IRS‐1 at Tyr896 is likely impaired in the metabolic syndrome subjects with fasting hyperphosphorylation of IRS‐1 at Ser636 and/or Ser337. Activation of GSK3 or JNK1 was not quantified in these subjects, but previously we reported that baseline phospho‐mTOR was not increased in metabolic syndrome subjects, although post resistance training the increase in mTOR activation was nearly twice that of the control subjects (Layne et al. 2011). Disappointedly, exercise training without weight loss did not diminish the basal level of IRS‐1 serine excess phosphorylation or improve insulin responsiveness. In our previous reports, resistance training increased strength in metabolic syndrome volunteers by 28% (Layne et al. 2011) and stationary bike training increased aerobic fitness (VO_2_max) by 16% (Stuart et al. 2013), but the fat mass averaged close to 20 kg and did not change.

## Conclusion

These data suggest that in metabolic syndrome muscle, serine phosphorylation of IRS‐1, the physiological negative feedback that normally turns off the insulin signal, is already partially in effect in many of these subjects before insulin concentrations are augmented in response to a meal or exogenous insulin.

## Acknowledgments

The authors wish to thank the exercise science students who participated in subject training and evaluations, nutrition interns who interviewed and counseled the subjects, and research nurse, Susie Cooper Whitaker, who was instrumental in coordinating the subject recruitment and studies.

## Conflict of Interest

None declared.
